# Long-Term Follow-Up of Cognition and Mental Health in Adult Phenylketonuria: A PKU-COBESO Study

**DOI:** 10.1007/s10519-017-9863-1

**Published:** 2017-08-03

**Authors:** Rianne Jahja, Francjan J. van Spronsen, Leo M. J. de Sonneville, Jaap J. van der Meere, Annet M. Bosch, Carla E. M. Hollak, M. Estela Rubio-Gozalbo, Martijn C. G. J. Brouwers, Floris C. Hofstede, Maaike C. de Vries, Mirian C. H. Janssen, Ans T. van der Ploeg, Janneke G. Langendonk, Stephan C. J. Huijbregts

**Affiliations:** 1 University of Groningen, University Medical Center Groningen, Beatrix Children’s Hospital, Groningen, The Netherlands; 2 Department of Clinical Child and Adolescent Studies & Leiden Institute for Brain and Cognition, Leiden University, Leiden, The Netherlands; 3 University of Groningen, Department of Developmental and Clinical Neuropsychology, Groningen, The Netherlands; 4 Academic Medical Center, Amsterdam, The Netherlands; 5 University Hospital Maastricht and Laboratory Genetic Metabolic Diseases, Maastricht, The Netherlands; 6 Division of Endocrinology and Metabolic Diseases, Department of Internal Medicine, University Hospital Maastricht, Maastricht, The Netherlands; 7 Wilhelmina Children’s Hospital, University Medical Center Utrecht, Utrecht, The Netherlands; 8 University Medical Center St Radboud Nijmegen, Nijmegen, The Netherlands; 9 Center for Lysosomal and Metabolic Diseases, Erasmus Medical Center, Rotterdam, The Netherlands; 10 Department of Clinical Child and Adolescent Studies, Leiden University, Wassenaarseweg 52, P.O. Box 9555, 2300 RB Leiden, The Netherlands

**Keywords:** Phenylketonuria, Executive functioning, Executive motor control, Mental health, Adults, Longitudinal

## Abstract

Cognitive and mental health problems in individuals with the inherited metabolic disorder phenylketonuria (PKU) have often been associated with metabolic control and its history. For the present study executive functioning (EF) was assessed in 21 PKU patients during childhood (T1, mean age 10.4 years, SD = 2.0) and again in adulthood (T2, mean age 25.8 years, SD = 2.3). At T2 additional assessments of EF in daily life and mental health were performed. Childhood (i.e. 0–12 years) blood phenylalanine was significantly related to cognitive flexibility, executive motor control, EF in daily life and mental health in adulthood (i.e. at T2). Patients with a greater increase in phenylalanine levels after the age of 12 performed more poorly on EF-tasks at T2. Group-based analyses showed that patients with phenylalanine <360 µmol/L in childhood and phenylalanine ≥360 µmol/L from age 13 onwards (n = 11) had better cognitive flexibility and executive motor control than those who had phenylalanine ≥360 µmol/L throughout life (n = 7), supporting the notion that phenylalanine should be below the recommended upper treatment target of 360 µmol/L during childhood for better outcome in adulthood. Despite some results indicating additional influence of phenylalanine levels between 13 and 17 years of age, evidence for a continued influence of phenylalanine levels after childhood on adult outcomes was largely lacking. This may be explained by the fact that the patients in the present study had relatively low phenylalanine levels during childhood (mean: 330 µmol/L, range: 219–581 µmol/L) and thereafter (mean Index of Dietary Control at T2: 464 µmol/L, range: 276–743 µmol/L), which may have buffered against transitory periods of poor metabolic control during adolescence and early adulthood.

## Introduction

Notwithstanding the prevention of intellectual disability by decreasing blood phenylalanine (Phe) concentrations, the neurocognitive and psychosocial outcomes of patients with phenylketonuria (PKU; OMIM 212600) are on average still below the level of their healthy counterparts. PKU is a rare inborn error of metabolism, with a mean prevalence of 1:10,000, that is characterized by deficient hepatic enzyme phenylalanine hydroxylase (PAH) activity. PAH normally helps to convert Phe into tyrosine (Tyr), which is the precursor to l-dopa and consequently dopamine (Blau et al. 2010; van Spronsen et al. 2017). Untreated PKU patients show high blood Phe levels and low to normal Tyr levels. High blood Phe levels facilitate the blood–brain barrier exchange of Phe at the expense of other large neutral amino acids into the brain including Tyr and tryptophan. Therefore, high blood Phe results in high brain levels of Phe that may affect white matter (Dyer 1999) and reduced brain availability of the precursors Tyr and tryptophan for the neurotransmitter synthesis of dopamine and serotonin, respectively. Clinically, untreated PKU is characterized by severe intellectual disability, neurological problems, motor deficits, and behavioural problems (Blau et al. 2010; DeRoche and Welsh 2008; Jahja et al. 2016; Smith and Knowles 2000). Treatment, through a Phe-restricted diet plus amino acid supplements, tetrahydrobiopterin (BH_4_, being a pharmacological chaperone protein of PAH) supplementation, or both, reduces blood Phe levels, which results in lower Phe levels and probably higher levels of neurotransmitter precursors such as Tyr and tryptophan (Blau et al. 2010; van Vliet et al. 2015, 2016). When started early after birth, treatment with dietary Phe restriction prevents intellectual disability, but, compared to healthy controls, PKU patients generally still have lower IQs and perform more poorly in several cognitive domains (Albrecht et al. 2009; Huijbregts et al. 2002a, c; Jahja et al. 2014; Moyle et al. 2007; Smith and Knowles 2000; Waisbren et al. 2007). The most frequently observed cognitive deficits in treated PKU are in executive functions (EFs), i.e. ‘higher-order cognitive abilities that control and coordinate behaviour and constitute the driving force of goal-directed behaviour’ (Christ et al. 2010; Huijbregts et al. 2002c, 2003; Moyle et al. 2007). Both cognitive impairment in treated PKU (Antenor-Dorsey et al. 2013; Blau et al. 2010; Huijbregts et al. 2002c) and internalizing mental health problems, such as anxiety, depression and mood swings (Anjema et al. 2011; Arnold et al. 1998; Cappelletti et al. 2013; Jahja et al. 2013; Smith and Knowles 2000; Weglage et al. 2000) have repeatedly been associated with concurrent and historical blood Phe levels. However, questions remain regarding long-term consequences of elevated Phe in various periods of life on cognitive functioning in adults with PKU.

The key statements of the first European guidelines on PKU clearly show the lack of valuable data on the relation between outcome and metabolic control from adolescence onwards (van Spronsen et al. 2017). While there are some studies relating adult outcomes to history of metabolic control, and other studies indicating the existence of certain sensitive periods for elevated Phe with respect to cognitive development (Huijbregts et al. 2002b), none of these provide complete and conclusive evidence. Studies with repeated measurements are very scarce. One longitudinal study reported on neurocognitive functioning of 57 adult patients aged 19–41 years who were re-examined after a 5-year interval (Weglage et al. 2013). Patients had a lower IQ than controls, but psychomotor function and sustained attention were not different between PKU patients and controls at either time point. The authors further distinguished between participants younger and older than 32 years old: older patients had slower information processing than controls at both time points. Group differences did not change during the 5-year interval. High blood Phe levels in childhood and adolescence were related to poorer IQ, information processing and attention in adulthood. A limitation of this study of Weglage et al. (2013) was that both assessments took place during adulthood, so cognitive development could not be mapped from childhood onwards. A second longitudinal study assessed 14 PKU patients in childhood and early adulthood. Patients were 8–14 years at time point 1 and 22–28 years at time point 2. That study showed that differences in neuropsychological outcome between patients and controls became smaller throughout adolescence and adulthood, but did not disappear (Nardecchia et al. 2015). Regarding lifetime Phe, specifically those with Phe between 501 and 600 µmol/L (n = 3) performed worse than those with lifetime Phe below 500 µmol/L (n = 4). These authors also differentiated between patients with Phe below or above 600 µmol/L during the interval between the two time points: those with Phe above 600 µmol/L (n = 7) during the interval had poorer cognitive outcome as adults compared to controls (n = 14). This study provided evidence for an influence of adolescent or ‘second decade of life’ metabolic control on adult cognitive outcomes. A limitation of this study was that the authors could not control optimally for childhood Phe levels in their statistical analyses, as Phe levels were only included for the first 4 years of life, whereas critical stages of EF-development occur later in childhood as well (Huijbregts et al. 2002b). So there is no strong evidence yet that adolescent or adult Phe levels truly influence adult outcome: it is still possible that childhood Phe levels, which are often related to Phe levels later in life, most strongly determine adult outcome.

The present study aimed to examine mental health, and development of executive functioning and executive motor control of PKU patients from childhood into adulthood in relation to historic metabolic control, distinguishing between different developmental stages and using different methodological approaches, compared to previous studies.

## Methods

### Participants

This study is part of the Dutch longitudinal multicentre PKU-COBESO study. Between February 1997 and October 1998, executive functioning and executive motor control were assessed in 67 children with PKU, aged 7–14 years (Huijbregts et al. 2002c). Patients that could be reached and who were willing to participate a second time were followed up between February 2012 and May 2015, at which point all of them were adults. At time point 1 (T1) and time point 2 (T2) identical tasks were performed, with additional tasks and questionnaires at T2 (Jahja et al. 2013). Patients were recruited through six Dutch university medical centres and on both occasions assessed in their treatment centre by the research staff. All patients were diagnosed by neonatal screening, which has been introduced in the Netherlands in September 1974, and treated early after birth. At both time points, T1 and T2, blood samples were taken on the day of neuropsychological testing in order to obtain concurrent Phe levels (conPhe). Historical Phe levels were collected from physicians’ databases. Indices of dietary control (IDC) were calculated as the mean of all half-year median Phe levels until the day of testing. Thus, IDC1 was computed from birth until the day of testing at T1, and IDC2 (i.e. lifetime Phe) represented Phe levels from birth until the day of testing at T2. To create a difference score between both time points, i.e. to determine the increase in Phe between the two assessments, IDC1 was subtracted from IDC2 (i.e. IDC difference score). Mean of half-year median Phe levels between 0 and 12 years, between 13 and 17, and ≥18 years were also calculated.

Furthermore, based on the question whether different upper target Phe levels should be maintained during childhood (0–12 years) and thereafter, and the most frequently recommended upper target Phe level of 360 μmol/L, patients were divided into groups: Phe <360 μmol/L in childhood and Phe <360 μmol/L from age 13 (‘low–low’ group); low Phe in childhood and high Phe ≥360 μmol/L from age 13 (‘low–high’ group); and high Phe ≥360 μmol/L in childhood and high Phe from age 13 (‘high–high’ group).

### Measures

The overall multicentre study (PKU-COBESO, Jahja et al. 2013) addressed cognitive, behavioural and social sequelae of early and continuously treated PKU patients in relation to history of metabolic control. A standardized testing protocol was used for each participant and took approximately 2.5 h to complete including breaks (Jahja et al. 2013). The following three computerized tasks of the Amsterdam Neuropsychological Tasks (ANT; De Sonneville 2014) were used to assess executive functioning and executive motor control on two different occasions, i.e. at T1 and T2.

In the Flanker (FL) interference task, which measures inhibitory control but more specifically interference suppression, stimuli consist of squares divided up into nine smaller squares. Participants must respond to the colour of the central smaller square, while ignoring the surrounding eight smaller squares (i.e. the flanker stimuli). When the central stimulus is blue, participants have to press the left mouse button; when the central stimulus is yellow, they have to press the right mouse button. In part 1 the flanker stimuli had a neutral colour (20 trials) or the same colour (20 trials, i.e. the compatible condition) as the central stimulus, while in part 2 the flankers had the same (40 trials, compatible condition) or the interfering (40 trials, incompatible condition) colour. The interfering colour meant to elicit a response with the other hand if it would have been the central stimulus colour (Huijbregts et al. 2002c). The differences in error percentage and reaction time (RT) between compatible and incompatible flanker stimuli in part 2 of the task were used to measure inhibitory control/interference suppression.

The shifting attentional set-visual (SSV) task measures inhibitory control but more specifically inhibition of prepotent responding, and cognitive flexibility. On the screen a horizontal bar consisting of ten squares is presented. In task part 1 (40 trials, compatible condition), participants follow a green block which randomly moves across the bar to left or to right: movements to the left require left mouse button presses and movements to the right require right mouse button presses. In part 2 (40 trials, incompatible condition), the randomly moving block is red and a response is required opposite to the direction of the movement, i.e. movements to the left require right mouse button presses and vice versa. In part 3 (80 trials), the block changed randomly into green or red after each movement and participants switch between the rules of task parts 1 and 2 (i.e. compatible and incompatible responding) depending on the colour of the block after each random movement (Huijbregts et al. 2002a). This task requires inhibition of prepotent responding, when in task part 2 a switch has to be made from the automatic (compatible) response mode in task part 1 to the incompatible response mode, and it requires cognitive flexibility, when in task part 3, one has to switch between the two active response modes based on the cue provided by the stimulus. The differences in error percentages and RT between part 1 and 2 represented inhibition of prepotent responding and the differences between part 1 and 3 represented cognitive flexibility.

Executive motor control was measured with the pursuit (PU) task. Participants had to follow an asterisk, which randomly moved across the screen, by placing the mouse cursor as closely as possible on top of the asterisk for 2 min, first with their non-dominant hand, then with their dominant hand (Huijbregts et al. 2003). Mean deviation from the moving target (i.e. accuracy of movement), and standard deviation of the trajectory that was followed (stability of movement) were used in analyses. A greater deviation or standard deviation indicated poorer executive motor control.

At T2, general/demographic information was collected and two questionnaires were administered. First, the Behavior Rating Inventory of Executive Function-Adult version (BRIEF-A) was used to measure executive functioning in daily life of adults (Roth et al. 2005). The questionnaire consists of 75 questions, assessing nine subdomains of executive functioning: Inhibit, Shift (Cognitive Flexibility), Emotional Control, Self-Regulation, Initiate, Working Memory, Plan/Organize, Organization of Materials, and Monitor. The subdomains Inhibit, Shift, Emotional Control and Self-Regulation together represent the Behavioral Regulation Index (BRI). Furthermore, the combined scores of the other five subdomains represent the MetaCognition Index (MCI). The Global Executive Composite (GEC) is the total score of all subdomains and represents the overall executive functioning in daily life. Continuous T-scores of the BRI, MI and GEC were used in statistical analyses. For descriptive purposes the following distinctions were used: participants have normal EFs when T-scores are below 50. A T-score between 50 and 65 is considered increased or borderline, and a T-score above 65 indicates clinical significance (i.e. in the clinical range).

Second, the Adult Self-Report (ASR) of the Achenbach System of Empirically Based Assessment (Achenbach and Rescorla 2003) measured mental health problems. It is a norm-referenced questionnaire consisting of 102 items with 3-point rating scales suitable for adults. Six DSM-IV-oriented scales are provided: Depressive problems, Anxiety problems, Somatic problems, Avoidant Personality problems, Attention Deficit/Hyperactivity problems, and Antisocial Personality problems. Next to these subscales, the overall internalizing, externalizing and total problem score were also used. For the three overall scales, a score below 60 is considered normal, scores between 60 and 64 are in the borderline range, and scores above 64 are in the clinical range. Thus, higher scores represent more mental health problems.

### Statistical Analyses

IBM SPSS Statistics 22nd version was used for statistical analyses. Associations between executive functioning and executive motor control at T1 and T2, and mental health at T2 on the one hand and several indicators of metabolic control on the other (i.e. conPhe at T2, IDC1, IDC2, IDC difference score, Phe 0–12, 13–17 and ≥18 years) were investigated using one-tailed Pearson correlations and partial correlations to control for Phe 0–12 years. Repeated measures analyses of variance were used to compare the ‘low–high’ groups at T1 and T2 on performance of the ANT-tasks measuring executive functioning and executive motor control. Z-scores of the EF measures at T1 and T2, calculated using age-appropriate performance scores from healthy controls in the PKU-COBESO study as norm reference, were used as dependent variables, where a low or negative Z-score indicated a better performance. Independent samples T-tests were conducted for comparisons of the ‘low–high’ groups on BRIEF and ASR scores at T2.

## Results

### Metabolic Outcome

Twenty-one patients (31% of the original cohort; 6 male, 15 female) completed a neuropsychological assessment at two time points. The remaining 69% of the original cohort was lost to follow up. Patients were not under treatment anymore and/or were out of sight from physicians (n = 40) or were not willing to participate (n = 6), usually due to time constraints. IDC at T1 (*t*(62) = 1.1, *p* = 0.29) and the pre-treatment Phe (*t*(64) = 0.4, *p* = 0.72) did not significantly differ between the 21 versus 46 patients, but conPhe at T1 was higher for the 69% that was lost (*t*(49) = 2.0, *p* = 0.027). However, data regarding executive functioning was gathered for both groups at T1. The two groups did not differ on inhibitory control (FL and SSV), cognitive flexibility (SSV) and executive motor control (PU), indicating that the two groups had similar executive functioning at T1.

For those 21 patients who participated at T2, mean age at T1 was 10.4 years (SD = 2.0) and 25.8 years (SD = 2.3) at T2 (see Table 1 for descriptive statistics). According to the traditional classification for biochemical subtypes within the hyperphenylalaninemias (Blau et al. 2011), four patients were classified as having hyperphenylalaninemia (HPA, i.e. a pre-treatment Phe level below 600 μmol/L), eight as mild PKU (pre-treatment Phe between 600 and 1200 μmol/L) and nine had classical PKU (pre-treatment Phe ≥1200 μmol/L). Mean conPhe at T1 was 346 μmol/L (SD = 204) while at T2 this was 719 μmol/L (SD = 351). Mean IDC at T1 was 312 μmol/L (SD = 96) and IDC at T2 was 464 μmol/L (SD = 138). Phe levels between 0 and 12 years, between 13 and 17 and ≥18 years are reported in Table 1. Correlations between Phe variables are displayed in Table 2. Phe concentrations significantly increased with age, as shown with repeated measures including Phe 0–12, 13–17 and ≥18 years (Wilks’*Λ* = 0.35, *F*(2,19) = 17.30, *p* < 0.001, *η²p* = 0.65). Within T1 and within T2, age did not significantly correlate with Phe. At T1, BH_4_ was not yet prescribed in the Netherlands. At T2, five patients used BH_4_ doses up to 20 mg/kg with a max of 1400 mg/day.

**Table 1:** Descriptive statistics PKU, and ‘low–high’ groups

	PKU = 21	Low–low = 3^a^	Low–high = 11	High–high = 7
Mean age T1 ± SD (range)	10.5 ± 2.0 (6.9–13.7)	11.0 ± 2.3 (9.3–13.6)	10.7 ± 2.1 (6.9–13.7)	9.9 ± 1.7 (7.0–12.4)
Mean age T2 ± SD (range)	25.8 ± 2.3 (21.0–30.5)	26.6 ± 2.5 (23.9–28.7)	25.7 ± 2.8 (21.0–30.5)	25.7 ± 1.7 (23.0–28.8)
Gender (male:female)	6:15	0:3	5:6	1:6
Socio-economic status: income	7 Above average	Average	5 Above average	2 Above average
Socio-economic status: education	14 Higher education	3 Higher education	7 Higher education	4 Higher education
IQ	102 ± 13 (71–120)	103 ± 14 (88–115)	103 ± 9 (92–120)	101 ± 18 (71–120)
Diagnostic Phe measurement	1300 ± 925 (120–3151)	205 ± 107 (120–325)	1252 ± 805 (450–3053)	1844 ± 904 (750–3151)
Biochemical PKU phenotype	4 HPA; 8 mild PKU; 9 classical PKU	3 HPA	1 HPA; 6 mild PKU; 4 classical PKU	2 mild PKU; 5 classical PKU
BH4 responsive	5 BH4 responsive	2 BH4 responsive	2 BH4 responsive	1 BH4 responsive
Concurrent Phe T1 ± SD (range)	346 ± 204 (30–860)	308 (2 missing)	257 ± 157 (30–475)	491 ± 211 (245–860)
IDC T1 ± SD (range)	315 ± 92 (192–548)	326 ± 53 (294–388)	252 ± 42 (192–327)	409 ± 84 (320–548)
Concurrent Phe T2 ± SD (range)	719 ± 351 (259–1550)	357 ± 87 (259–427)	807 ± 403 (336–1550)	735 ± 241 (345–1000)
IDC T2 ± SD (range)	464 ± 138 (276–743)	291 ± 15 (276–306)	424 ± 79 (322–547)	602 ± 112 (446–743)
IDC difference score (IDC2 minus IDC1)	149 ± 120 (−82–330)	−35 ± −41 (−82 to −6)	172 ± 93 (52–322)	194 ± 112 (−6–330)
Phe 0–12 years ± SD (range)	330 ± 91 (219–581)	308 ± 35 (286–348)	270 ± 40 (219–331)	434 ± 71 (380–581)
Phe 13–17 years ± SD (range)	533 ± 246 (267–1069)	293 ± 30 (268–326)	475 ± 172 (272–764)	728 ± 269 (267–1069)
Phe >18 years ± SD (range)	651 ± 258 (226–1105)	253 ± 23 (226–267)	658 ± 202 (465–1068)	809 ± 211 (528–1105)

*BH4* tetrahydrobiopterin, *IDC* index of dietary control, *PKU phenotype* based on diagnostic Phe measurement, *HPA* hyperphenylalaninemia, Phe 120–600 µmol/L, *mild PKU* Phe 600–1200 µmol/L, *classical PKU* Phe >1200 µmol/L

^a^The low–low group was excluded from statistical analyses because the sample size was too small

**Table 2:** Pearson correlations between indicators of metabolic control

	Concurrent Phe at T1	IDC at T1	Concurrent Phe at T2	IDC at T2	IDC difference score	Phe 0–12 years	Phe 13–17 years	Phe ≥18years
Concurrent Phe at T1	1.000							
IDC at T1	0.543**	1.000						
Concurrent Phe at T2	0.221	−0.117	1.000					
IDC at T2	0.529**	0.520**	0.570**	1.000				
IDC difference score	0.170	−0.174	0.747***	0.751***	1.000			
Phe 0–12 years	0.553**	0.933***	0.041	0.693***	0.078	1.000		
Phe 13–17 years	0.387^+^	0.234	0.488*	0.877***	0.830***	0.466*	1.000	
Phe ≥18 years	0.385^+^	0.170	0.778***	0.876***	0.879***	0.328+	0.754***	1.000

*p* < 0.05 values are indicated in bold

*IDC* index of dietary control, *T1* time point 1 in childhood, *T2* time point 2 in adulthood

*p < 0.05 **p < 0.01 ***p < 0.001,^+^ *p* < 0.10 (1-tailed)

Regarding the ‘low–high’ groups, three patients were allocated to the ‘low–low’ group (mean Phe <360 μmol/L when 0–12 years and from age 13 onwards), 11 patients were in the ‘low–high’ group (Phe <360 μmol/L until age 12 and ≥360 μmol/L from age 13) and seven patients were in the ‘high–high’ group (Phe ≥360 μmol/L in childhood and onwards). None of the patients had high values as a child and low values as an adult. Descriptive statistics and metabolic measurements for these groups are displayed in Table 1. Because the first group was too small, only the ‘low–high’ and ‘high-high’ groups were included in group-based statistical analyses. The ‘low–high’ group consisted of one patient with hyperphenylalaninemia, six patients with mild PKU and four patients with classical PKU, while the ‘high-high’ group included two mild PKU patients and five patients with classical PKU. The pre-treatment Phe concentration (*t*(16)= −1.5, *p* = 0.17) and the PKU classification (*χ* ^*2*^ = 2.3, *p* = 0.31) did not differ between the two groups. The socio-economic status, i.e. education and yearly income, of these two groups was similar, respectively *χ* ^*2*^ = 3.1, *p* = 0.37 and *χ* ^*2*^ = 1.7, *p* = 0.89.

### Participant Characteristics: Relationships, Education, Occupation, Income

Sixteen out of 21 patients (76%) had a long-term, romantic relationship, 13 have had two or more long-term, romantic relationships in the past. All patients completed high school. Eight patients (38%) followed or completed higher vocational education and six patients (29%) completed higher education (bachelor’s or master’s degree), which is comparable to the healthy Dutch population, according to the Dutch Central Bureau for Statistics (CBS 2016a). All patients had an occupation at T2, working 12–40 h/week. Seven patients (33%) had a higher than average income and 12 out of 21 (57%) were house owners of private property, similar to the Dutch population (CBS 2016b).

### Associations Between Metabolic Control, Executive Functioning and Mental Health

Regarding inhibitory control/interference suppression (FL-task), partial correlations (controlling for Phe 0–12 years) showed that Phe 13–17 years, the IDC difference score and IDC2 were significantly associated with percentage errors at T2 (see Table 3 for associations between Phe and ANT-tasks). The positive correlation between the IDC difference score and error percentage indicated that with a larger increase in Phe between childhood and adulthood, poorer performance was observed at T2.

**Table 3:** Partial correlations (Pearson, 1-tailed) between Phe and ANT

	*r*	*p*
Flanker—inhibitory control/interference suppression		
% errors T2		
Phe 13–17 years	**0.578**	**0.004**
IDC difference	**0.533**	**0.008**
IDC T2	**0.436**	**0.027**
Shifting attentional set-visual—cognitive flexibility		
% errors T2		
IDC difference	0.393	0.053
Phe 13–17 years	**0.426**	**0.039**
IDC T2	0.354	0.075
Reaction time T2		
IDC T1	**0.446**	**0.028**
Phe 0–12 years	**0.429**	**0.033**
Pursuit—executive motor control		
Mean deviation T2		
IDC T1	**0.490**	**0.012**
Phe 0–12 years	**0.404**	**0.035**
Standard deviation T2		
IDC T1	**0.550**	**0.005**
Phe 0–12 years	**0.515**	**0.008**

*p* < 0.05 values are indicated in bold

*Note* only significant correlations and (non-significant) trends shown

*IDC* index of dietary control, *T1* time point 1 in childhood, *T2* time point 2 in adulthood

Inhibition of prepotent responding (SSV-task) was not associated with any of the Phe indices. When measuring cognitive flexibility, the percentage of errors at T2 was significantly correlated with Phe 13–17 years while the correlation with the IDC difference score just failed to reach significance after controlling for Phe 0–12 years. RT at T2 was associated with IDC1 and with Phe 0–12 years.

Regarding executive motor control (PU-task), IDC1 and Phe 0–12 years were significantly related to accuracy and stability of executive motor control at T2. These correlations indicated that high Phe in childhood were related to poorer executive motor control in adulthood.

The Behavioral Regulation Index (BRI) of the BRIEF-A which was only measured at T2, was significantly associated with IDC1, Phe 0–12 years and IDC2 (see Table 4 for correlations between Phe and questionnaires). When controlling for Phe 0–12 years, the correlation with IDC2 became non-significant. The Global Executive Composite (GEC) was also related to Phe 0–12 years. These results demonstrate that childhood Phe is important for executive functioning in daily adult life.

**Table 4:** Partial correlations (Pearson, 1-tailed) between Phe and behavior ratings

	IDC T1		IDC T2		Phe 0–12 years		Phe 13–17 years		Phe ≥18 years	
	*r*	*p*	*r*	*p*	*r*	*p*	*r*	*p*	*r*	*p*
BRIEF-A										
Behavioural regulation index	**0.454**	**0.025**	**0.415**	**0.039**	**0.501**	**0.014**	0.173	0.239	0.288	0.116
Global executive composite	0.329	0.084	0.361	0.064	**0.397**	**0.046**	0.233	0.168	0.264	0.138
ASR										
Depressive problems	0.346	0.073	**0.402**	**0.044**	**0.456**	**0.025**	0.322	0.090	0.266	0.135
Anxiety problems	0.255	0.146	0.213	0.191	0.289	0.115	0.151	0.268	0.076	0.378
Somatic problems	**0.609**	**0.003**	**0.451**	**0.026**	**0.721**	**0.000**	0.247	0.153	0.226	0.177
Avoidant personality problems	0.383	0.053	−0.038	0.439	0.349	0.072	−0.230	0.172	−0.134	0.292
Attention deficit/hyperactivity problems	**0.567**	**0.006**	0.351	0.070	**0.572**	**0.005**	0.169	0.244	0.211	0.193
Antisocial personality problems	**0.545**	**0.008**	**0.488**	**0.017**	**0.496**	**0.015**	0.182	0.277	**0.398**	**0.046**
Internalizing problems	**0.512**	**0.012**	0.130	0.298	**0.568**	**0.006**	0.007	0.488	−0.096	0.347
Externalizing problems	**0.586**	**0.004**	**0.538**	**0.009**	**0.619**	**0.002**	0.348	0.072	0.368	0.060
Total score	**0.524**	**0.011**	0.342	0.074	**0.593**	**0.004**	0.174	0.238	0.162	0.254

*p* < 0.05 values are indicated in bold

*IDC* index of dietary control, *T1* time point 1 in childhood, *T2* time point 2 in adulthood, *BRIEF-A* behavior rating inventory of executive function-adult version, *ASR* adult self-report

With respect to mental health at T2, Depressive problems were related to Phe 0–12 years and to IDC2. Somatic problems had a significant relation with IDC1, Phe 0–12 years and IDC2. IDC1 and Phe 0–12 years were associated with Attention Deficit/Hyperactivity problems. Antisocial Personality problems were related to IDC1, Phe 0–12 years, Phe ≥18 years and IDC2. Overall internalizing problems were associated with IDC1 and Phe 0–12 years. The externalizing problem scale was related to IDC1, Phe 0–12 years and IDC2. However, when controlling for Phe 0–12 years, the correlations with Phe indices after childhood became non-significant. Finally, the overall total problem scale was again associated with IDC1 and Phe 0–12 years. The IDC difference score was not significantly related to the ASR scores.

### Multiple Testing

Regarding the neuropsychological test outcomes, correction for multiple testing (seven IDCs times eight outcome measures) would result in non-significant correlations only, as a *p* value of 0.0009 would be the criterion. We opted for calculating all these correlations as the different IDCs may provide (subtly) different information. Still, the IDCs were often strongly correlated (see Table 2). If one would retain only IDC at T1 and the IDC difference score, as they represent childhood Phe and Phe-change thereafter, and therefore seem the most meaningful indices, no significant correlations are retained either, as a *p*-value of 0.003 would be the criterion. Some could be retained by reducing the number of outcome measures, e.g. one per task. However, there is no evidence to support one should choose either RT, error rate, or stability of performance as main outcome measures of the tasks.

Questionnaire data showed stronger correlations with IDCs, with IDC1 and Phe0–12 correlations with the BRI of the BRIEF almost remaining significant after applying *p*-level corrections with a factor 4, i.e. two indices of metabolic control (i.e. IDC1 and IDC difference score or Phe 0–12 and Phe >12), and two outcome measures (MCI and BRI indices). A number of results obtained on the ASR actually did remain significant after applying corrections with a factor 12 (i.e. two IDCs, six ASR-dimensions): somatic problems, attention deficit/hyperactivity problems, or 4 (i.e. two IDCs, two ASR-dimensions): internalizing and externalizing problems. The pattern of correlations indicated that especially those with IDC1 or Phe 0–12 were robust (Table 4). Further group comparisons were made (see below) in order to link results to upper target Phe levels (for different age periods) according to current treatment guidelines, and to gain more insight into development of cognition and mental health in PKU patients relative to healthy controls.

### Comparison ‘Low–High’ and ‘High–High’ Groups

**Fig. 1:**
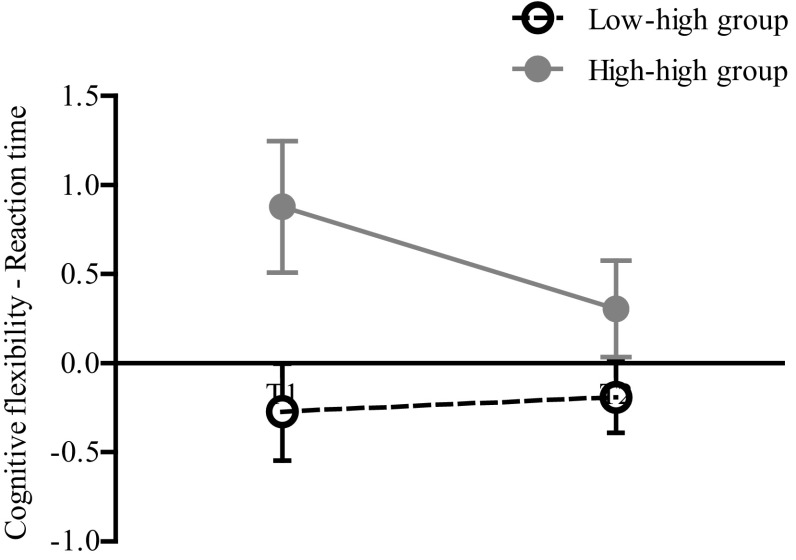
Reaction time of cognitive flexibility. Group effect was significant: at both time points the low–high group performed faster than the high–high group

**Fig. 2:**
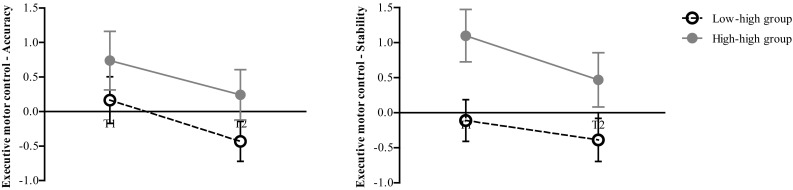
Executive motor control: accuracy and stability of movement. Time effect was significant for accuracy and stability: all participants improved over time. Group effect was significant for stability only: the low–high group had a more stable executive motor control than the high–high group

For inhibitory control/interference suppression (FL-task), repeated measures analyses of variance did not show significant effects for time or group. Also, there was no interaction effect. On the SSV-task, there were no significant effects for inhibition of prepotent responding. Concerning cognitive flexibility, the ‘low–high’ group was significantly faster than the ‘high-high’ group at both time points (*F*(1,15) = 8.0, *p* = 0.013, *n* ^*2*^_*p*_ = 0.349) (see Fig. 1). Although descriptive statistics showed better Z-scores on cognitive flexibility for all patients at T2 compared to the age-appropriate norm, time and interaction effects were not significant. For executive motor control (PU-task) there was a significant effect for time. All participants improved over time compared to the age-appropriate norm: they had a more accurate (*F*(1,16) = 5.5, *p* = 0.033, *n* ^*2*^_*p*_ = 0.255) and stable (*F*(1,16) = 4.5, *p* = 0.050, *n* ^*2*^_*p*_ = 0.219) executive motor control at T2. Also the group effect was significant for stability of movement: the ‘low–high’ group had a more stable executive motor control than the ‘high–high’ group (*F*(1,16) = 5.6, *p* = 0.031, *n* ^*2*^_*p*_ = 0.259) (see Fig. 2).

Regarding the BRIEF scales on executive functioning in daily life, there were no statistically significant differences between the ‘low–high’ (n = 11) and ‘high–high’ (n = 7) groups. Finally, with respect to mental health as measured by the ASR scales, the ‘high-high’ group reported significantly more Somatic problems than the ‘low–high’ group (*t*(14) = −3.8, *p* = 0.002). When examining the descriptive statistics, two patients from the ‘low–high’ group (18%), scored in the borderline range of the internalizing scale and had a lifetime Phe of 420 and 514 µmol/L respectively. Two patients (29%) from the ‘high-high’ group were in the clinical range with lifetime Phe of 446 and 678 µmol/L. The latter patient with the highest Phe also scored in the clinical range of the externalizing and overall total problem scale. This patient also scored in the clinical range of the BRIEF. The patients in the normal range had similar lifetime and childhood Phe as those in the borderline and clinical range.

## Discussion

The present study provided evidence for long-term effects of elevated childhood Phe on cognitive functioning and mental health during adulthood, although specifically for a number of cognitive indices more recent Phe levels, particularly Phe level between 13 and 17 years of age, were also associated with outcome, even after controlling for childhood Phe. Results confirmed that Phe levels increase with age. Phe levels increased in adolescence and were the highest in adulthood. This is consistent with results of previous studies (e.g. Walter et al. 2002; Walter and White 2004) and may be due to the fact that recommended treatment targets are higher in adolescence and adulthood compared to childhood (Blau et al. 2010; Weglage et al. 2013). A larger increase from childhood to adult Phe levels was associated with poorer inhibitory control/interference suppression and cognitive flexibility, as measured with the ANT, during adulthood.

Examining the changes in outcome between childhood and adulthood showed that executive motor function improved over time for PKU patients relative to the norm reference of healthy controls, whereas this was not observed for inhibitory control and cognitive flexibility. These last results are in line with findings from the study of Weglage et al. (2013), who concluded that in 5 years’ time cognitive performance of adult patients remained stable, despite an increase in Phe levels. The present study showed that such stability of relative deficits apparently extends over a period of 10–15 years. In line with findings from Nardecchia et al. (2015) we showed a significant improvement relative to healthy controls in executive motor control between childhood/adolescence and adulthood. Nardecchia et al. (2015) reported relative improvements for other cognitive domains as well, incorporating a period between test occasions that was almost as long as ours, but in a sample that was smaller than ours. Differences may be related to measurement tools and sample characteristics. Our sample, for instance, consisted predominantly of patients at the higher end of the socio-economic status spectrum. Moreover, the 21 patients with T2 assessments did not differ significantly from healthy controls at T1 on the FI- and SSV-tasks, which is in contrast with findings reported for the entire PKU sample at T1 (Huijbregts et al. 2002a, c). Even though the 21 patients did not differ from the 46 patients lost to follow-up regarding EF at T1 either, the evidence indicating that the T2 PKU group is relatively “well-off” or relatively mildly affected is more convincing than the evidence showing a lack of differences between patients that did versus those that did not participate at T2. This should be considered when interpreting our study results.

An explanation for the stability or improvement of cognition among PKU patients in both studies might lie in good or good enough treatment compliance and subsequent low Phe levels after childhood. The fact that the mean Index of Dietary Control at T2 (i.e. lifetime Phe level) was 464 µmol/L, with a range of 276–743 µmol/L seems to support this notion. Despite the fact that Phe levels increased after childhood, these may still have been sufficiently low to allow patients to ‘close’ the gap with their healthy counterparts or at least not worsen compared to them. Considering this indicator of metabolic control, further patient characteristics, e.g. on education, employment, and romantic relationships, and the fact that this selection of patients did not differ from controls at T1, it cannot be ruled out either that our findings, and those of Nardecchia et al. (2015) and Weglage et al. (2013), are positively biased and that this bias extends to domains beyond metabolic control.

Relatively good cognitive and psychosocial development of individuals with PKU over longer periods of time may depend upon their childhood Phe levels. Unfortunately, this could not be comprehensively studied by Nardecchia et al. (2015) who only used Phe from the first 4 years of life as covariate for the negative association between lifetime Phe and executive functioning whilst critical periods for EF-development are known to occur beyond the age of four (Huijbregts et al. 2002b). This is particularly unfortunate as we found evidence for the importance of childhood Phe levels (i.e. between 0 and 12 years) with two different statistical approaches. First, we investigated whether Phe levels from different developmental stages were differentially associated with our neurocognitive and mental health outcome measures. Results showed that generally childhood Phe as measured by IDC at T1 or Phe between 0 and 12 years was solely or more strongly associated with the outcome measures at T2 (i.e. in adulthood). With two exceptions regarding higher-order executive functioning, which is known to continue development in the second decade of life, associations with post-childhood Phe levels were non-significant after controlling for childhood Phe. Second, we created groups of PKU patients based on Phe levels during childhood and thereafter, and compared outcomes during adulthood. As a cut-off for group assignment we took a Phe level of 360 μmol/L, which is the recommended upper target Phe level worldwide for children up to the age of 12 (van Spronsen et al. 2017; Vockley et al. 2014), and, in the USA, also for the period thereafter (Vockley et al. 2014). As there were no patients going from high levels during childhood to low levels during adolescence and beyond, and only three patients in the ‘low–low’ group, we compared two groups: the ‘low–high’ group (Phe <360 μmol/L until age 12 and ≥360 μmol/L from age 13) and the ‘high–high’ group (Phe ≥360 μmol/L throughout life). The ‘low–high’ group performed better on a number of tasks than the ‘high–high’ group, specifically with respect to cognitive flexibility and executive motor control, and also had better mental health outcomes as adults. These findings suggest that those patients who had low Phe in childhood had better outcome in adulthood than those who already had high Phe in their first 12 years of life.

In our study, adult mental health problems were also exclusively or most strongly related to elevated Phe in childhood. Phe between 0 and 12 years was related to Depressive, Somatic, Attention Deficit/Hyperactivity, Antisocial Personality problems, and the overall internalizing, externalizing and total problem scale. Associations between mental health problems and later Phe indices were no longer significant after controlling for childhood Phe. Brumm et al. (2010) demonstrated a relationship between severity of behavioural symptoms and timing and degree of exposure to Phe. They concluded that children with high Phe in earlier years were more likely to be affected and had more severe mental health problems. Our results are consistent with their finding.

It may be considered a limitation of this study that many statistical tests have been carried out. After correction for multiple testing by using adjusted *p* values many significant results were lost. However, a pattern of results remained evident indicating a larger influence of childhood Phe levels (Phe 0–12 years, IDC1, Phe low–high vs. Phe high–high) compared to Phe levels later in life. Also, there are a number of arguments supporting the use of multiple statistical tests. Generally, two broad sets of analyses have been used: correlations between indices of metabolic control and outcome measures, and group-based analyses involving comparisons between PKU patients with Phe levels below the recommended upper target limit as a child (0–12 years, below 360 µmol/L) and higher levels thereafter and PKU patients with Phe levels higher than 360 µmol/L throughout life. Although the correlations were corrected for childhood Phe levels, which already made results indicative of the relative influence of Phe during different stages of life, the added value of the group-based analyses is that they relate possible differential influences of Phe during different stages of life to treatment guidelines (i.e. the upper target Phe level of 360 µmol/L). The different IDCs that were included in the correlational analyses were often relatively strongly related, but it was still considered important to investigate not only IDC until T1 and until T2 but also to distinguish Phe levels between 0 and 12, 13 and 17 years and for 18 years and older because these periods are distinguished in treatment guidelines as well. Furthermore, results showed a potential influence of change in Phe levels between childhood and thereafter that could not be captured by analyses using absolute Phe levels. Thus, it may be concluded that results of different tests were complementary. Another, related limitation of the present study is that strong evidence for further specificity in the strengths and weaknesses profile of PKU patients cannot be provided. For this, we would have to include even more tasks (assessing RT and error rate) and (informant-rated) questionnaires, preferably in a larger sample. In general, however, clear and converging evidence for the continued influence of childhood Phe on adult cognitive (EF-) and behavioural outcomes was provided.

The most important limitation of our study is probably that our sample seemed positively biased and therefore not entirely representative of the “average” adult PKU patient. Because we missed many patients who had participated as a child, we also had a small sample size. This would render the outcome of further comparisons unreliable, e.g. between BH_4_ users and non-users, or a group-based analysis including the ‘low–low’ group. Whereas considering the rarity of PKU the sample size is still acceptable and comparable to other studies using a follow-up design in PKU, future studies should seek for ways or strategies to enhance more comprehensive inclusion of patients. Another concern may be the inclusion of different subtypes of PKU, making this sample heterogeneous. However, this enabled us to examine the effect of lower Phe and this was taken into account when analysing the ‘low–high’ and ‘high–high’ groups: there was no difference in type of classification between the two groups. A different type of limitation lies in the choice of Phe levels to represent metabolic control in different stages of development. As noted, we used 0–12, 13–17, ≥18 years, to differentiate between different developmental stages. However, the exact age ranges representing different developmental and possibly critical stages for cognitive and psychosocial development are unknown. Whereas we made informed choices and examined Phe levels during different time windows as well (e.g. the average Phe level between IDC at T1 and IDC at T2, which had a correlation of approximately *r* = 0.9 with Phe 13–17 years and similar correlations as Phe 13–17 years with outcome measures), more general knowledge on developmental milestones or critical developmental stages for EF and psychosocial functioning would be very helpful in this respect. A final limitation lies in the possible underestimation of their own mental health problems by PKU patients. Although it is relatively custom to use self-reports in order to assess mental health, a more comprehensive assessment (using other informants and/or expert interviews) would be advisable for future studies.

In summary, our study showed that executive functioning in PKU patients is mostly stable over time from childhood into young adulthood, with some improvements relative to controls observed as well. Furthermore, long-term effects of elevated childhood Phe on cognitive and mental health outcome later in life were observed, supporting the notion that childhood Phe should be below the recommended treatment target of 360 µmol/L for better outcome in adulthood. Although the influence of childhood Phe levels was generally the strongest, the extent of the increase in Phe levels after childhood and Phe levels between 13 and 17 years of age were also related to some aspects of adult EF. With regard to this, we cannot entirely rule out the influence of later Phe levels as participants were almost exclusively characterized by good metabolic control from birth onwards and had grown up in social and socio-economic environments that may be considered protective or beneficial for cognitive and psychosocial development.
